# Ten Simple Rules to becoming a principal investigator

**DOI:** 10.1371/journal.pcbi.1007448

**Published:** 2020-02-20

**Authors:** John S. Tregoning, Jason E. McDermott

**Affiliations:** 1 Department of Infectious Disease, St Mary’s Campus, Imperial College London, London, United Kingdom; 2 Biological Sciences Division, Pacific Northwest National Laboratory, Richland, Washington, United States of America; 3 Department of Molecular Microbiology and Immunology, Oregon Health & Sciences University, Portland, Oregon, United States of America; Whitehead Institute for Biomedical Research, UNITED STATES

## Introduction

The biggest choke point in an academic career is going from postdoc to principal investigator (PI): moving from doing someone else’s research to getting other people to do yours. Being a PI is a fundamentally different job to being a postdoc; they just happen to be in the same environment. It is not an easy transition. It draws on few of the skills you learn at the bench, and the odds are clearly not ever in your favor. So, calling this article Ten Simple Rules is obviously a simplification. It is more accurate to call them ten tricky steps.

In this article, we use PI to mean anyone who runs their own research group using funding that they have been awarded to answer their own questions. PI encompasses a number of different job titles depending on where the research is performed: fellow, lecturer, reader, associate professor, and senior scientist. One test is whether you can describe the people working for you as the X group, in which X is your surname. The normal route from undergraduate to lab head involves a PhD, one or more postdoc positions, and then PI. Given the diversity of ways to be a PI, the final step up from postdoc takes a number of forms. In the United Kingdom, this tends to be either an individual fellowship or a lecturer position, and in the United States, it generally starts with an independent position with associated funding—either as a start-up package or funded grant.

The aim of this article is to identify some of the broader skills (rules 1–4) and behaviors (rules 5–10) that can help with getting a PI position. It is meant as advice not instruction. As you will see, we are advocating the development of social intelligence, which is as useful in the world outside academia as within it.

## Rule 1: Have ideas

**Fig 1:**
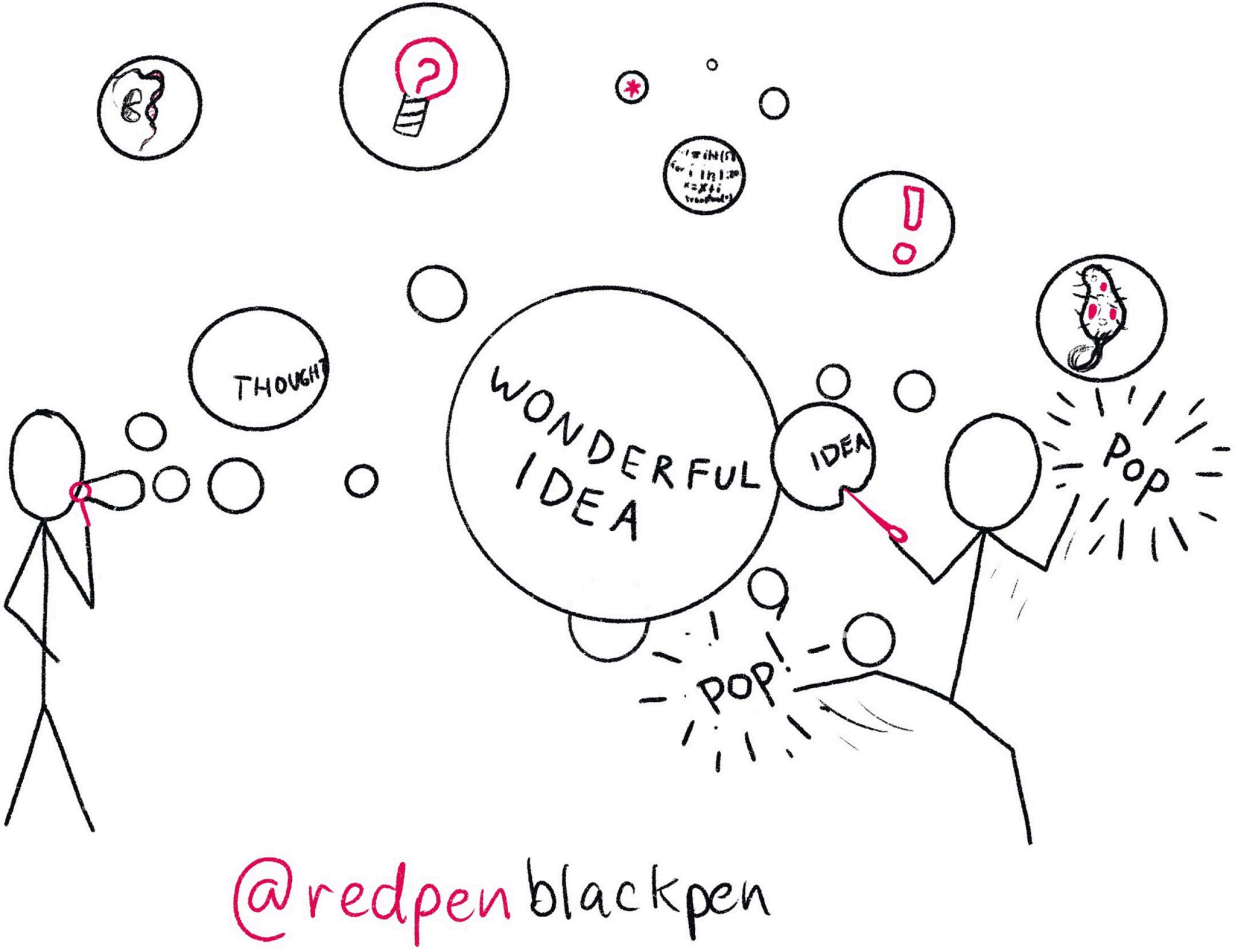
Have ideas. Ideas in the scientific space take a while to nucleate, but are ephemeral, like bubbles. When fully formed, good ideas will survive scrutiny and questioning. But be careful about exposing them to others too early. Criticism, however well-meaning, can burst half formed ideas.

Creativity is central to being a PI—seeing new connections, thinking of new ideas, and using current understanding to develop future plans. Unfortunately, creativity is incredibly nebulous and can feel at odds to the scientific process (Fig 1). Be receptive to ideas whenever they come, especially as they often come at the most inconvenient of times—when dropping the kids off or at 4:00 in the morning. Find ways to capture these flitting ideas. Accept that there are few truly novel ideas: Reading around will provide you with inspiration for your own problems. Whilst it is more about making things than doing science, *Every Tool’s A Hammer* captures what it is to be creative [1].

**Fig 2:**
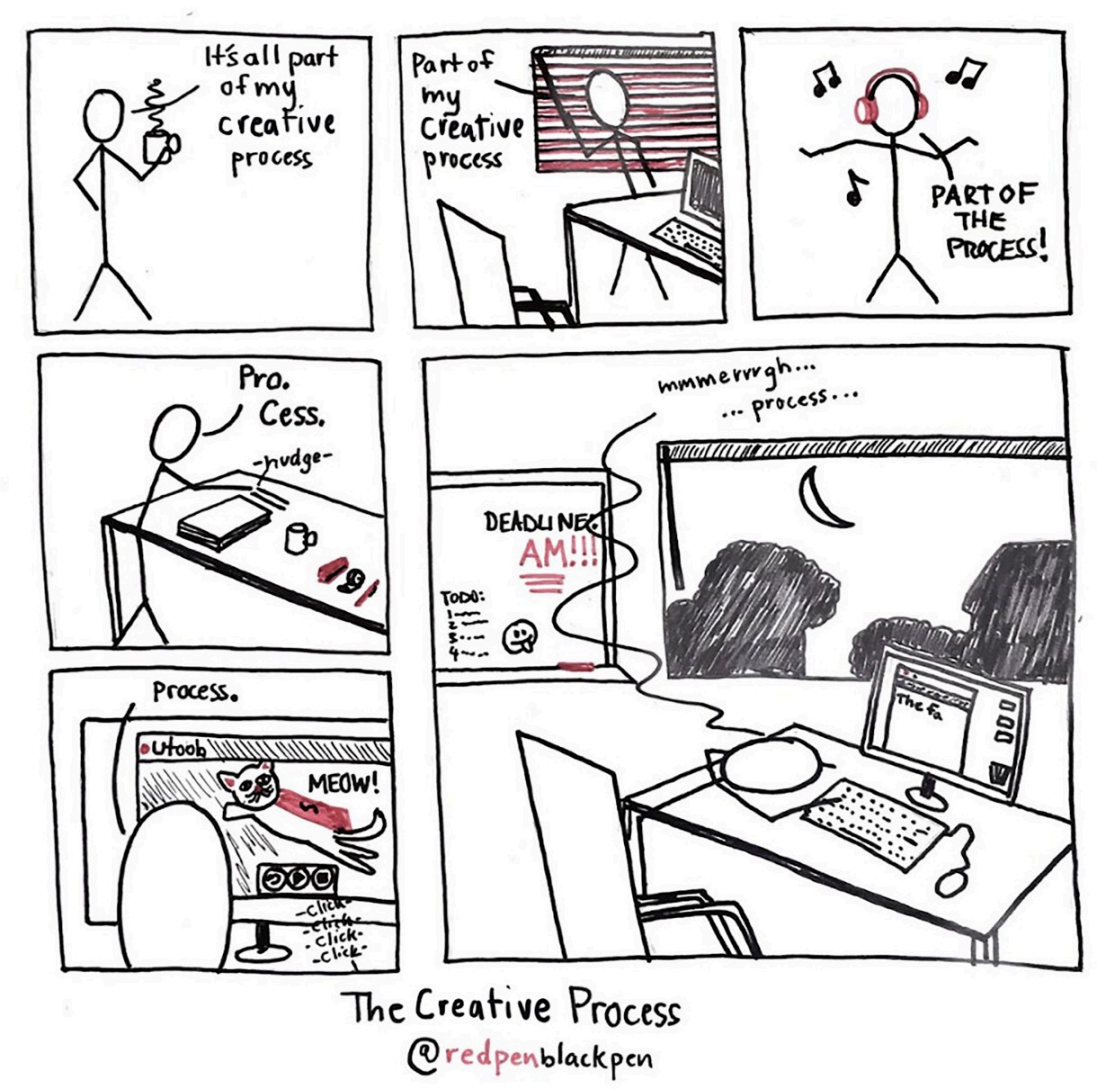
The creativity process. Writing is a part of the job, and it is important to have a process that helps you write (and good time-management skills around that process as well).

Learn to accept that ideas do not just come by themselves and allow them time to develop: It is entirely normal to have more bad ideas than good ones. Even if the net product from the day is a waste bin full of paper and some tea-stained scribbles—having a creative process, whatever that is, is very important (Fig 2). At some point, these scribbles do turn into ideas, but there is no magic wand. One analogy is of a nucleation point: initially there is a swirling mass of ideas with no form, and eventually they coalesce into something. Caffeine helps.

Discussing the idea with other people is vital, not just to shape the idea but also to find the right home for it. However, the timing of discussion is critical. Ideas are really fragile: Feedback at early stages will often focus on negatives, not the potential, and many a good idea has ended up in the bin due to early “help.” Stephen King advises developing the initial idea with the door closed and only opening the door when the idea is mostly formed [2].

## Rule 2: Publish papers

**Fig 3:**
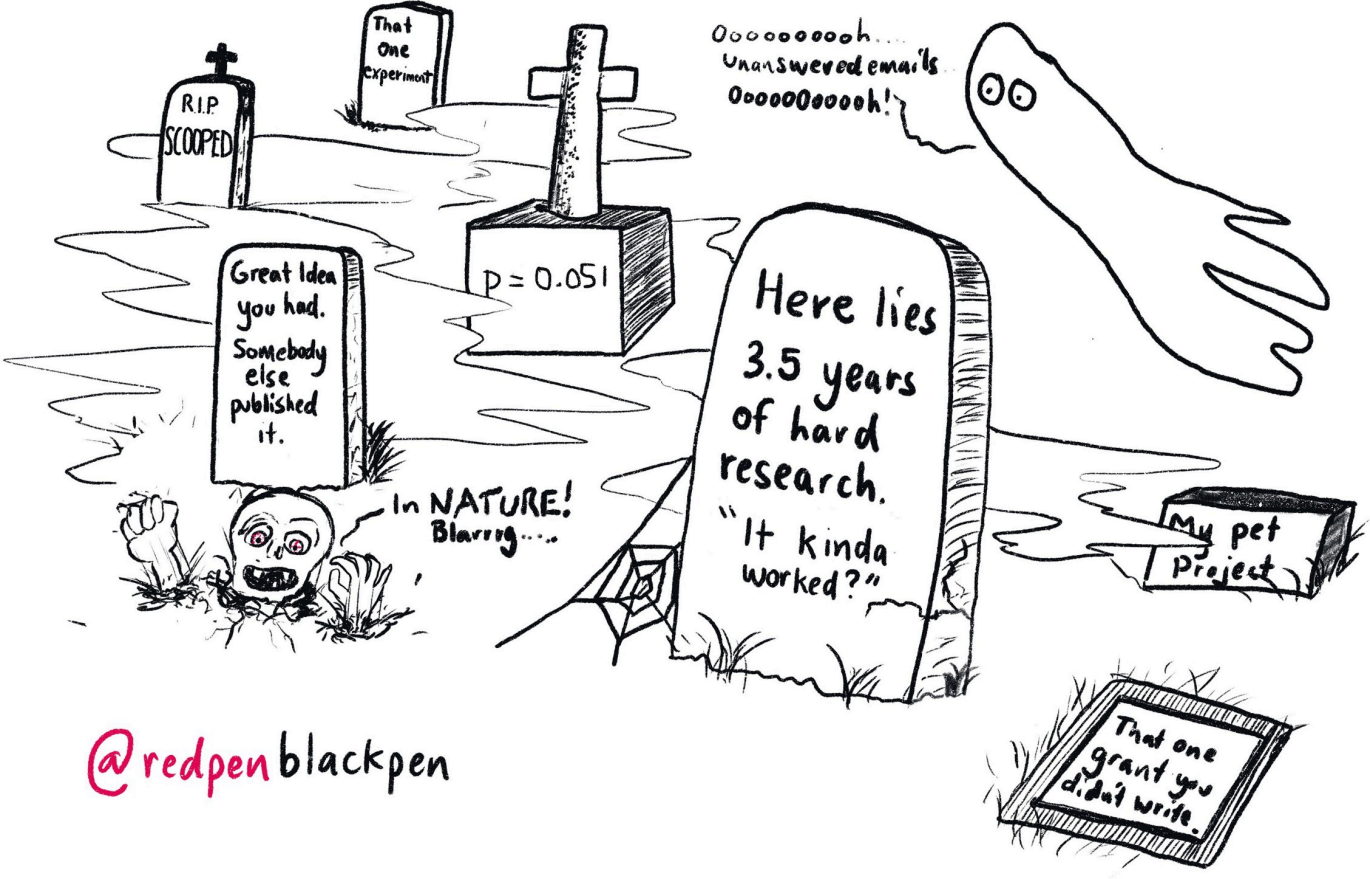
Publish papers. Every successful PI has a “graveyard” of uncompleted and/or unsuccessful ideas. The trick is to have a lot of things going forward to make sure some survive.

Have no illusion: The main thing you need on your curriculum vitae (CV) is papers (Fig 3), preferably first-author papers and ideally first-author papers in which you are the corresponding author, with the occasional last author paper thrown in for good measure. Papers are both the imprint we leave on the scientific world and the genealogy by which other people can track our pedigree. A recent analysis identified papers as the single most important factor in getting tenure [3].

While first-author papers are the gold standard, you also need breadth and depth in your publications. Look for opportunities to contribute to other people’s work. Coauthored papers are important; they demonstrate an ability to collaborate as part of a team and can lead to roles in larger grants. One approach is to take advantage of what Dr. Tregoning calls “top drawer specials.” Nearly every PI will have projects that never quite make it, due to people leaving or research priorities changing. Often all that is required is for someone to pull preexisting data into paper form. Completing these side projects whilst waiting for your main project to bear fruit is a very efficient way of CV boosting and practicing your writing.

You also need some evidence that you can get grants. Unfortunately, getting grants can feel like a catch-22 for early career researchers. You cannot become a PI till you get grants; you cannot get grants until you become a PI. This is a tricky but not insurmountable problem. There are some smaller pots of money that you can apply for, including travel grants and (sometimes) internal funding schemes. At the very least, be involved in grant writing. Learn the process, so it doesn’t come as a horrible shock. If you do contribute to a grant from your current lab, ensure that you are named on it in some role.

Whilst there is no way that you can get an academic post without papers, papers alone are not sufficient: There are many people with great CVs and no tenure. There are other skills and behaviors that you need.

## Rule 3: Research what the job involves and learn to juggle

**Fig 4:**
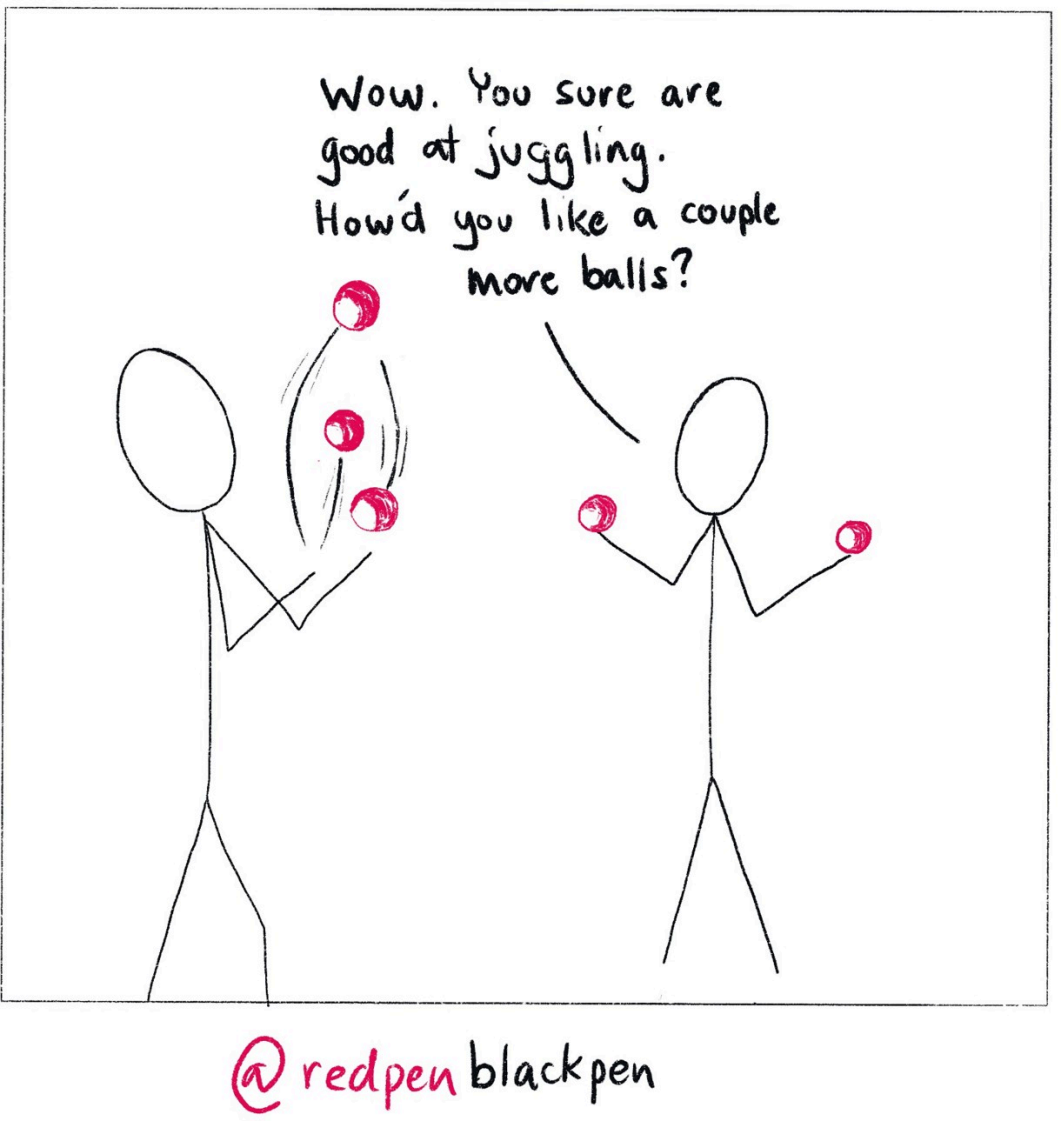
Juggling, always juggling. As a lab head, your duties may stretch your abilities to accomplish things. Be aware that if you are viewed as successful, you will be asked to take on even more.

Before losing sleep about not becoming an academic, understand what an academic career involves. Spoiler alert: It is mostly juggling (Fig 4). Before becoming a PI, Dr. Tregoning drew heavily from fiction to form vague and entirely wrong ideas of the role, with elements of Hogwarts, Jordan College in *Lyra’s Oxford* (from *His Dark Materials*), and the *Jurassic Park* cloning lab (before the dinosaurs escaped). However, a closer parallel is that you are an entrepreneur running your own business within an organization that provides some core support services.

As the head of a lab, you are responsible for fundraising, fund managing, purchasing materials and equipment (some of which is extremely specialist, even unique), training and managing staff working with dangerous materials, publicizing the current research, and planning future research. As a teacher you are expected to inspire and educate the next generation with a range of teaching styles that are appropriate for either 300 students in a lecture or for a single student, as a mentor. On top of this, you are expected to help with the administration of a large complex organization with upwards of 10,000 staff. Hiring good people can help to distribute some of this load. Dr. Hope Jahren in *Lab Girl* captures much of the joy and pain of an academic career [4].

No two academic careers are the same. This is one of the best aspects of the job. We are given (some) flexibility to choose our own routes. Whilst there are core elements—teaching, research, and administration—the make-up of each person’s role can be very different. This will vary by individual and institution: Some places are research only, and some are focused on teaching. Spend some time thinking about what type of academic you might want to be and where your strengths lie so you can best prepare. But also try everything out: You might discover a previously unknown talent for steering committees, designing curricula, or community outreach.

## Rule 4: Develop your skills

The skills you learn in the lab during your PhD and postdoc are by and large irrelevant to those necessary to run a lab. While you will get your next job based on your CV (your previous employer and your publications), you will only succeed in your next job based on your ability to do a range of other things [5]. The biggest difference is lab and technical work. As a PI, the amount of time you spend doing raw science (be it in a wet lab, in a field, or at a computer) dramatically decreases. This can be tricky to come to terms with, but as the leader of the group, your main responsibility is to support your team. Invest some time in developing skills outside the lab. To get more of a sense of the skills needed to run a lab, read *At the Helm* [6].

The most important skill is learning to write well. The time that you no longer spend generating data is quickly filled by time writing grants and papers. Writing science well is not trivial. There are many resources that can support you in learning to write, including Stephen King’s *On Writing* [2], Roy Peter Clark’s *Writing Tools* [7], and Joshua Schimel’s *Writing Science* [8]. There are also academic articles—including some excellent 10 Simple Rules [9]. If you do not have time to read these, take George Orwell’s advice from “Politics and the English Language” and never say anything that is outright barbarous [10]. Nothing beats practice and feedback. Bear in mind, there are other ways to present your ideas [11].

The other critical skill is learning how to work with people. Get management experience before you go live with your own lab: That way, your early mistakes don’t affect you long term. The easiest way is to do this is outside science, which can come in many forms—working in a shop, volunteering at a shelter, running a children’s football team, or even joining the army [12], which may seem a bit extreme, but it gives you a chance to explore what works and what doesn’t. Working with students is another rewarding way of developing your management skills. Likewise, ask your postdoc mentor if you can take on management responsibilities in your current lab.

## Rule 5: Focus on the prize

**Fig 5:**
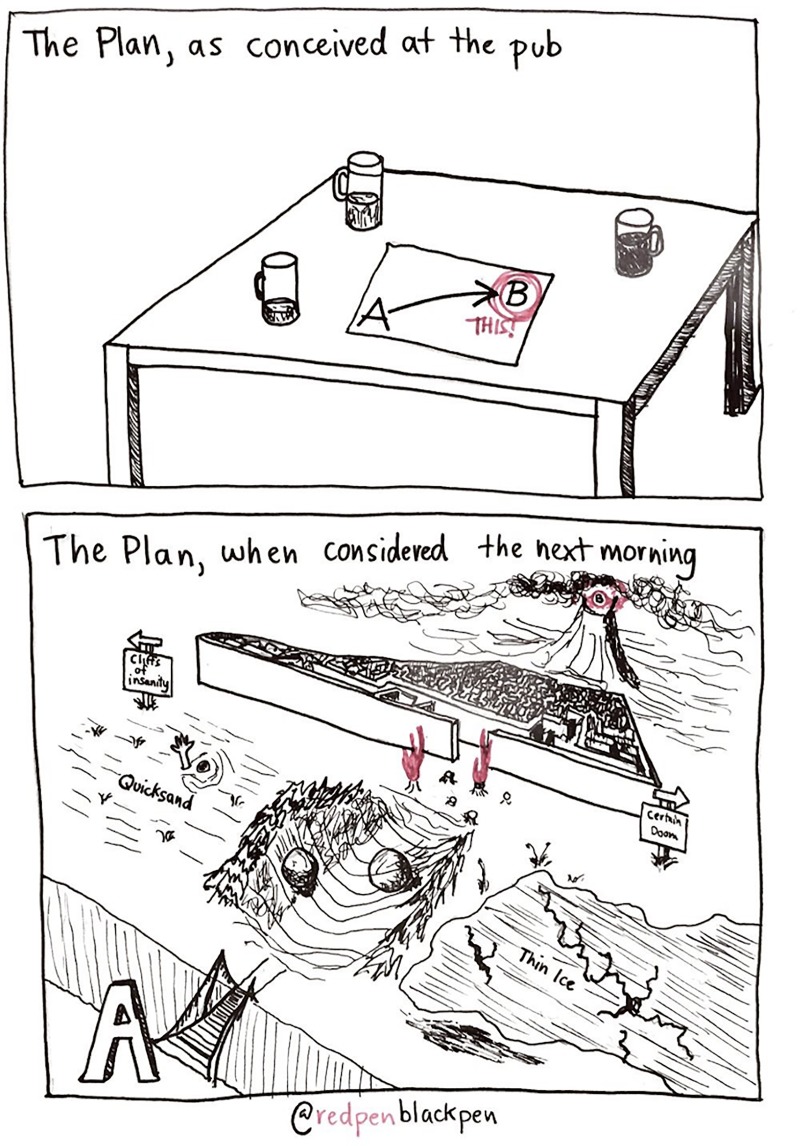
Focus on the prize. Generally, your plan seems a lot more straightforward than the way things actually happen. It’s important to remain flexible about how you achieve your goals and, indeed, what those goals actually are. But also remember that other people’s paths were not necessarily as smooth as they appear.

A lot of becoming a PI boils down to attitude: A major defining quality is relentless perseverance in the face of the odds. Your initial plan about how you’re going to get there is usually a lot simpler and easier than the course you will eventually take, but don’t give up (Fig 5). Whilst you have limited hours in the day and, unfortunately, a limited time from getting your PhD to getting onto the tenure track, the solution isn’t only working harder. Focus on the things that help you cross the line. In order to do this, you need to identify what these key things are and be able to evaluate the benefit per unit of time invested.

**Fig 6:**
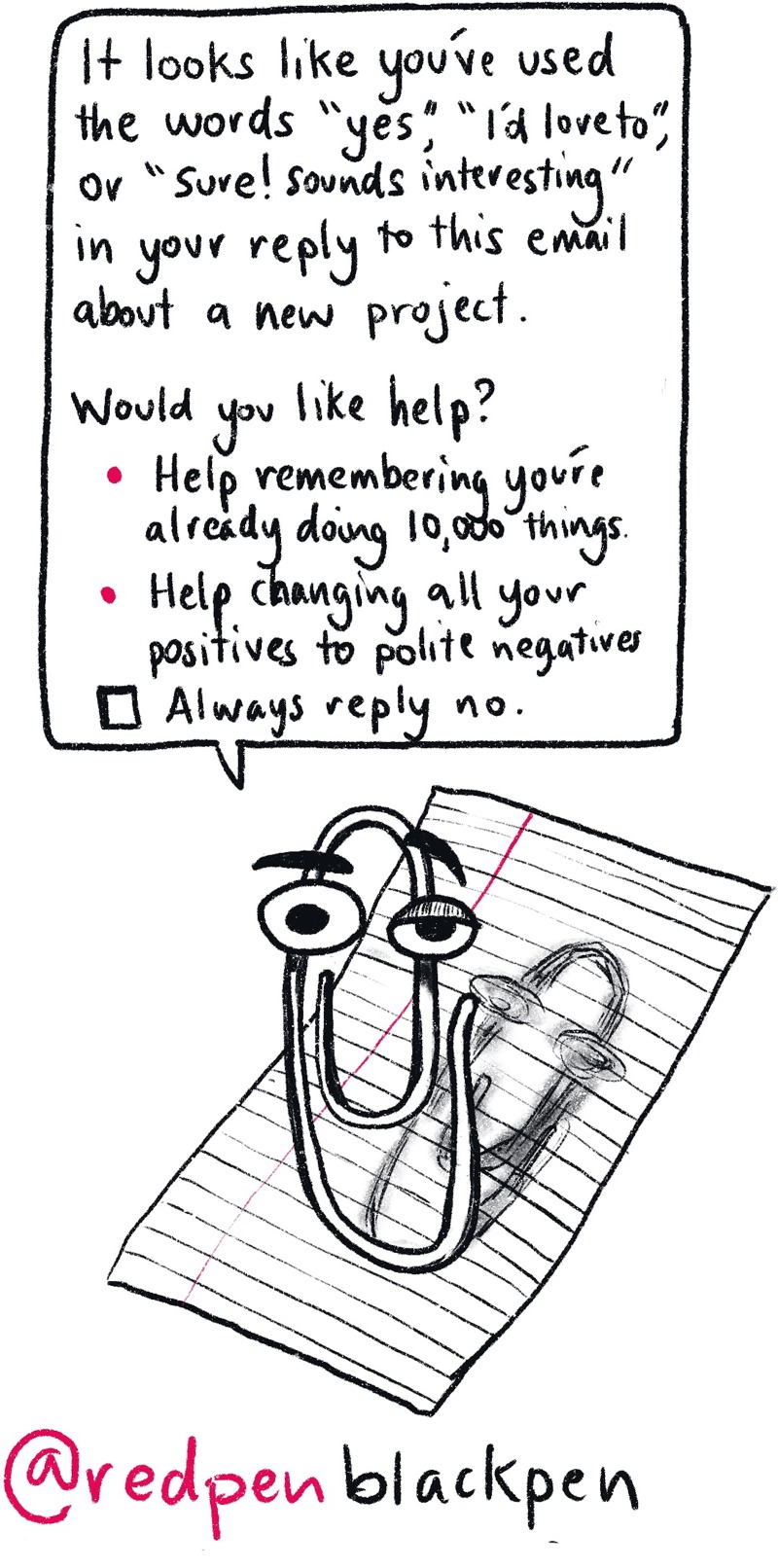
Just say no. The ability to say “no”—even when the opportunity seems exciting—is a must for PIs.

Make the most of your time: Think about what you are being asked to do and how it helps you become a PI. For example, be selective about the work you do: Don’t start any work unless you can see the route to publication. One of the major skills is learning to say no, even if the opportunity is really shiny (Fig 6). This can be tricky when working for someone else who has ten scatty ideas that go nowhere for every one that makes the final draft. You have to be choosy and occasionally say no if the project looks like a dead end. Of course, there is a fine line between being self-driven and self-centred; you still need to do things that contribute to the smooth running of the lab you find yourself in. This extends into faculty positions. Being collegiate makes you more employable: No one wants to work with “that person” with a reputation for selfishness.

Be aware that what others show to the outside world isn’t the whole truth. Professor Alice Prince at Columbia recently described how her National Institutes of Health (NIH) biosketch was a poor reflection of the person she is [13]. Going from a successful PhD studentship in a big lab to a postdoc in an even bigger lab, followed by a fellowship, with *Cell*, *Nature*, and *Science* (CNS) papers every step of the way is still perceived as the only route to being a PI. But very few people take this route; doing a muddle of short-term contracts is a much more realistic route. Trust us: It is possible to get a PI job without publishing in *Nature* (the authors submit their Google Scholar profiles as evidence: JST and JEM).

## Rule 6: Bounce back from failure

**Fig 7:**
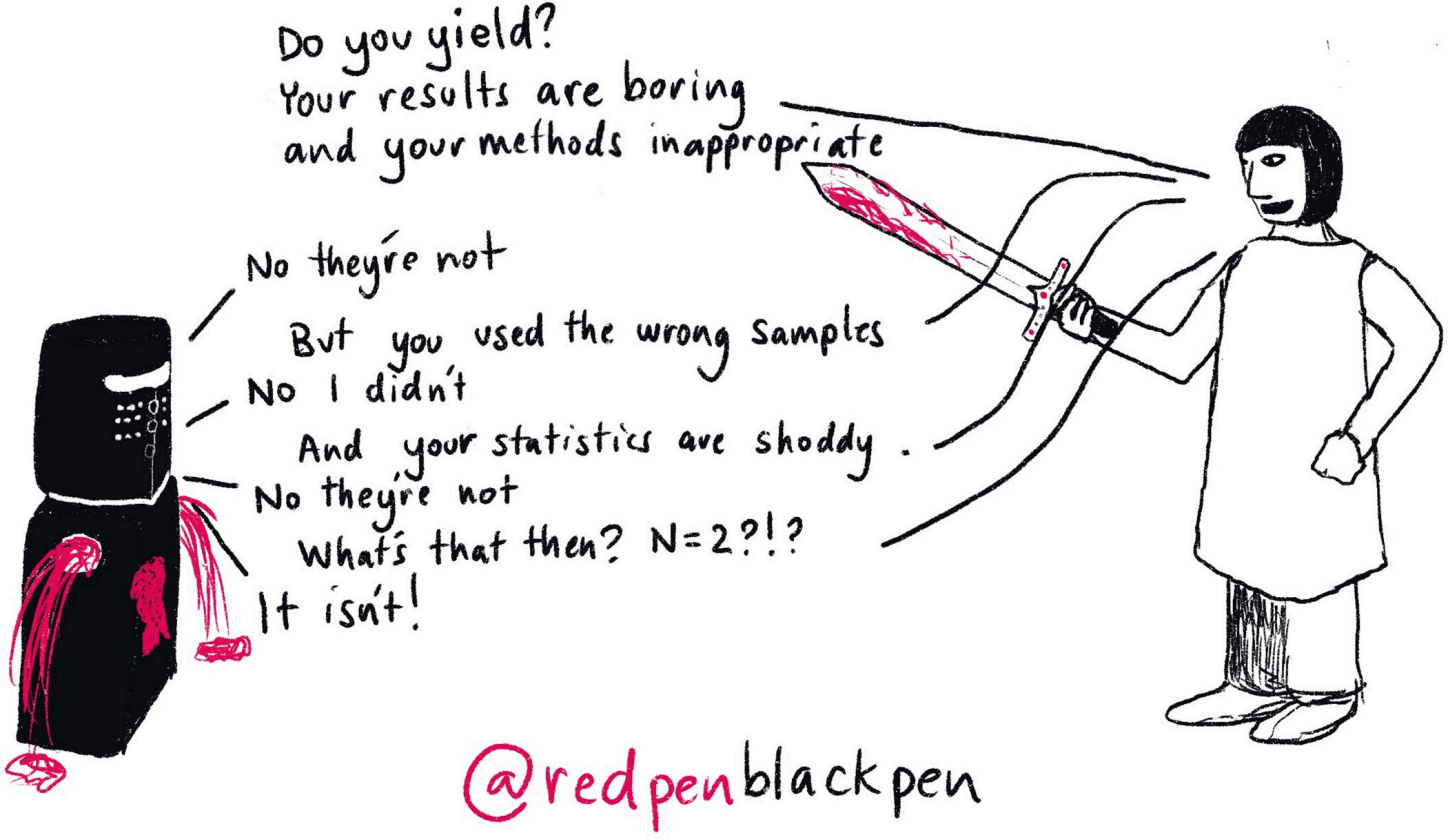
Bounce back from failure. Persistence and perseverance are two qualities that are vital to becoming and being a PI. You should stand up for and argue your point but remain aware that you could be wrong.

No matter how focused you are, you are going to fail. One of the most common experiences of being an academic is failure. You will fail on your path to becoming a PI, and you will fail once you become a PI. It is not the failing that matters; it is how you bounce back again (Fig 7). No one succeeds all the time; to use a sporting analogy, Babe Ruth had a batting average of 0.342—meaning he missed the ball 65% of the time—Lionel Messi requires 5.79 shots per goal, and Serena Williams misses 40% of her first serves. Likewise, a PI who gets more than 20% of their grants funded is a superstar. Learning coping strategies is vital. Some of the things that help are as follows:

### Reflective practice

Carol Dweck’s brilliant book, *Mindset* [14], can help you to fail better. She suggests reframing failure as a learning opportunity. After the initial mourning period, look again at rejected papers and grants in the light of the reviewer’s feedback and see what you can improve.

### Revise, recycle, and resubmit

Any single rejection is not the end of the idea. Many grant applications require a resubmission. In 2015, NIH R01-grant success rate was at approximately 30% for resubmissions, compared with 10% for original submissions [15]. And even if your grants are not funded, there are ways to get the work done, chopping them into new projects or running them on the side of other funded things. Likewise, no single journal is perfect, and with the San Francisco Declaration on Research Assessment (DORA) advocating for a shift from impact factor, it is worth considering on what other grounds your work will be evaluated [16].

### Learn to roll with reviewers’ punches

Flawed as it is, the peer-review system is the least worst, and we are stuck with it for now. Remember, reviewers don’t reject your work because they hate you. Stand in the reviewer’s shoes: They have to make decisions on a large number of grants from a multitude of subjects in limited time. Sometimes, your work may not make the cutoff, and coming back a second time with new data may be enough to get you over the line.

### Depersonalize it

It is important to separate your personal worth from your successes and failures at work. Failure and the resultant iterations and corrections are part of creativity. Depersonalizing failure allows you to accept constructive criticisms and move both your ideas and yourself forward [17].

## Rule 7: Develop your brand

We have two things to sell, our ideas and ourselves. Of the two, the main product we sell is ourselves, which (at work at least) is defined by our CV: what we have worked on and who we have done it with and where. Develop a single memorable “personal brand,” which can be used when meeting potential collaborators, conference organizers, and funders. Have a single line “elevator pitch” that summarizes what you do, backed up with an exciting case study. The brand includes the types of research you aspire to do and the initial projects you might run. The hope is that by pitching this brand successfully you will be at the forefront of people’s thoughts when they are putting together grants, consortia, or seminars. Your brand could even help you end up in front of that elusive tenure-track or lectureship appointment committee. Part of this brand development is identifying your strengths and honing them. Whilst you shouldn’t ignore your weaknesses, your strengths are the foundation on which you build your career. The brand is no longer limited to papers and conferences. It is possible to reach whole new audiences through social media [18]—though be aware the boost in connectivity may not compensate for the time lost down the rabbit hole.

## Rule 8: Believe in yourself

**Fig 8:**
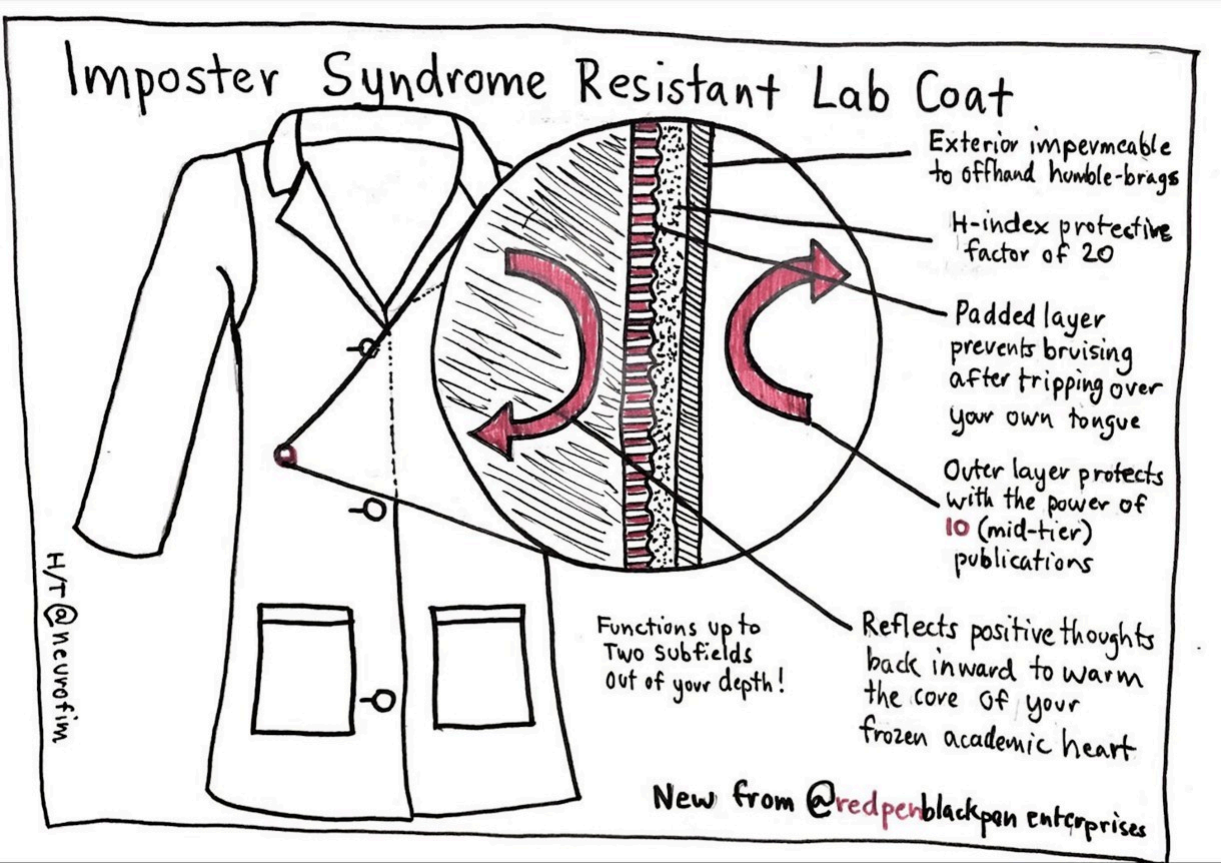
Believe in yourself! You can make one of these lab coats for yourself by trying to remember that everyone in science experiences imposter syndrome to some extent and that questioning yourself, your perspective, and your position is actually an essential part of the scientific process.

Developing your brand is easier said than done, in part because of the curse of imposter syndrome [19], in which you doubt your own talents and fear that you will be revealed as a fraud (Fig 8). Nearly everyone in academia suffers from it to some degree or other. The process of peer review is a major contributor: You and your work are routinely judged by others and, given the high failure rate, are going to be found wanting, often.

At the end of the day, academia is just a job, a fascinating and fun job that can occasionally take up every single second you have but ultimately just a job. There is no value in becoming a PI at any cost. Ensure you live a life outside work, for your own mental health and for that of your team. Academic burnout is very real; it is okay to take a break to reset stress levels: for example, Dr. Tregoning runs [20]. Sometimes stepping back a bit can even help in terms of creativity and headspace. Likewise, if you work every hour of the day to become a PI, there is no spare capacity when life inevitably doesn’t run smoothly.

## Rule 9: Build a network of mutually supportive people

Networking is central to being a PI. The best way to do this is to meet people face to face: Get out there and break bread. Carry business cards at all times. Go to conferences, consortia, and congresses: Plan who you want to meet at the conferences, even email them in advance to arrange time at the meeting. Often, smaller conferences can give you better chances to meet people. But be aware that talks aren’t the only part of the conference; the social events are great places to meet people and to learn. Networking isn’t limited to networking up; network sidewise with your peers and down with the people who you are training. Virtual networking can help: This paper is the offspring of a Twitter conversation.

The other consideration is choosing the right boss and environment to work in. The ideal boss is supportive, enabling, and generous in credit. If you can’t find that, find someone who will let you get on with things independently so you can develop your own ideas. At the very least, avoid bosses who will wittingly or unwittingly damage your career. Try to discover what flavor a potential boss might be before committing to work for them; discrete questions when you visit a lab for an interview can be helpful.

**Fig 9:**
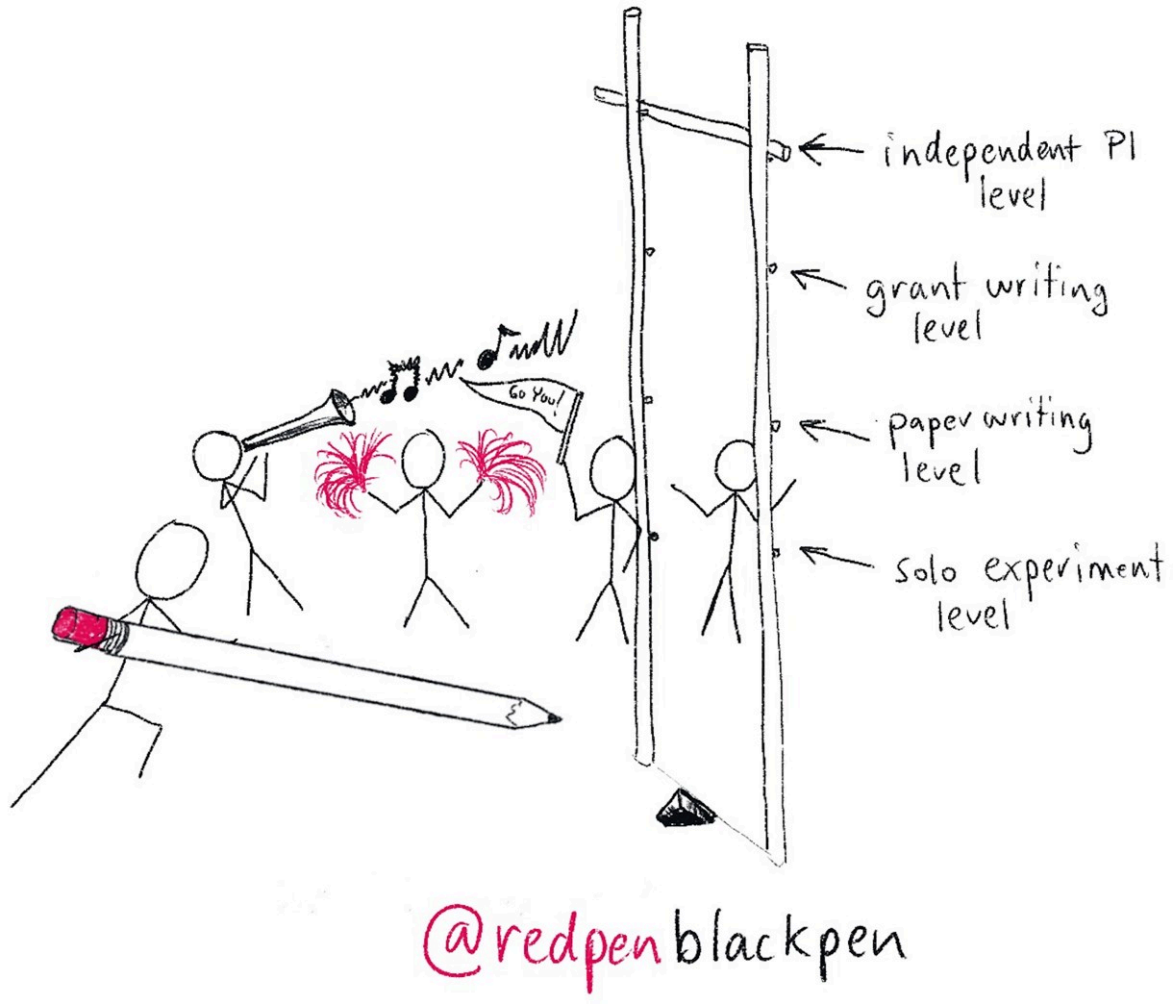
Get help. Remember that sometimes you need the cheering section and sometimes you are the cheering section for others. PI, principal investigator.

Ultimately, nobody can succeed on their own (Fig 9). There are many functional reasons to build up a network of people: Other people have different skills, expertise, and access to equipment or reagents. Many of the best things that happen are often random offshoots from chance meetings, for example, papers that sprang from discussions with the external examiner at a viva or collaborations formed at conference bars.

But, more importantly, network because working on your own is rubbish, boring, and sad. Nurture your colleagues at every step of the way. Be kind wherever possible [21]. You could probably succeed by pursuing a divide and conquer, winner takes all attitude, and you could probably name people who have done this. But don’t; as then, everyone loses.

## Rule 10: Know when to quit

Disclaimer: Even if you follow these 10 rules, there is no guarantee you will end up running your own lab. Knowing when to cash out is as true for scientific dead-ends as career ones. Chasing after something long after it has eluded you is not going to add to your overall life happiness.

Remember that academia isn’t the only job. There is the perception that becoming a PI is the one true path. As a PhD student, it is easy to say that your dream goal is to become a PI. This is mostly groupthink resulting from the lack of visible alternatives. Within the university system, the most visible individuals who are successful are the PIs; the people who are succeeding in other careers have by necessity left the university system. Becoming a PI is merely one career path amongst several that are available to scientifically trained graduates, all of which will value the skills you have developed along the way. Make sure that in upskilling you consider employability outside the sector or giving yourself an edge within the sector; this is part of the reason the authors started blogging and drawing: It was a new thread drawn from skills we enjoyed that gave us a different dimension [22].

Deciding when enough is enough is the hardest rule and is in direct conflict with Rule 6 about learning to fail. This is a decision only you can make, but don’t rush it as it is not unusual to want to quit often.

## Conclusion

It has not been easy to condense how to become a PI into Ten “Simple” Rules, particularly when there are so many tensions—being focused on yourself without being selfish, being resilient in the face of failure but knowing when to quit, and gaming it without being cynical. Notable absentees from this list are technical expertise, hard work, and knowledge of the field: These are a given, but there are more people who are hardworking, skillful, and knowledgeable than there are PI jobs. There is clearly a role for luck, but you need to be prepared to exploit opportunities.

Ultimately, if becoming a PI is what you want to do, do not let anyone (including yourself) put you off. Yes, the odds are against you, with a recent study reasserting the low rate of postdocs who become tenure-track faculty [23], but there are jobs with worse, steeper pyramids. Tracking the likelihood of reaching the top in other careers is one way to normalize academia: for example, acting—of the 300 million people in the US only 51,000 people work as actors (Actors’ Equity Association figures 2017). Likewise, while nearly every child in England plays soccer at some point in their childhood, only 22 of them make the national teams. Compared to these horrific odds, academia is relatively easy: 15% of the roughly 20,000 postdocs employed in the US will end up in a tenure-track academic position [24].

Ultimately, it is social intelligence (sometimes referred to as ‘soft skills’) that can make the critical difference. The good news is that you are already developing a lot of these skills by stealth: Time management, working with people, and juggling priorities are all part of being a postdoc. Even better, these leadership skills—being more resilient, being kind, looking after yourself and your colleagues, and focusing on your goals—apply to all jobs. So even if your academic aspirations don’t play out, you will be in a position to succeed in any role.
